# Supplementary material for: A theory that predicts behaviors of disordered cytoskeletal networks
Source: Mol Syst Biol. 2017 Sep 27;13(9):941. doi: 10.15252/msb.20177796 (PMC5615920; doi:10.15252/msb.20177796)
Supplement: Supplementary file 1 — Appendix [file MSB-13-941-s001.pdf]

A theory that predicts behaviors of disordered cytoskeletal networks

Julio Belmonte, Maria Leptin and François Nédélec

Appendix

We describe here first the details of the networks studied and the hypotheses under which our research was conducted. We then summarize the approach and specific parameters of simulations used to verify the theory, which are based on previously published methods and Open Source software (Cytosim). Finally, to illustrate both the theory and the simulations, we present the analysis of a system where bi-functional motors and passive crosslinkers act on a network of stabilized filaments. For this example, the theory makes a quantitative prediction of how the contraction rate depends on the numbers of connectors acting between the filaments.

Table of contents:

A. General Assumptions
B. Description of the Simulations
C. Extraction of the Contraction Rate from Simulations
D. Calculation of the Connector Subunits Probabilities
E. Prediction of Contraction Rates for Various Connector Mixtures
F. Prediction of Contraction Rates with End-Binders
G. Contraction Rate for Networks of Semi-Flexible Filaments
Appendix Table S1 – Simulation Parameters
Appendix Table S2 – Biological Parameters
Appendix Table S3 – Cytosim Configuration File
References

A. General Assumptions

We describe here the general hypotheses of our study. In brief, we considered idealized disordered networks of semi-flexible polar filaments, simplified to retain only those elements that appear essential for contraction. Filaments are infinitely thin, and their degrees of freedom represent position, orientation and bending, but longitudinal extension and twist around the axis are ignored. A network is made of thousands of filaments, positioned and oriented randomly in all directions thus constituting an isotropic and uniform meshwork. The filaments have a fixed length and no assembly or disassembly occurs at their ends. All the filaments have the same length and this length is shorter than the diameter of the network. We ignore edge effects as much as possible, to focus on the average behavior deep within the network.

The **filaments** are either *rigid* or *semi-flexible*, meaning that the length of the filaments is shorter than their persistence length. We expect this condition to hold true for cytoskeletal networks encountered in reality, since the persistence length is ~18μm for F-actin and ~5000μm for microtubules. We assume that the network is initially free of strain, implying that since they are shorter than their persistence length, the filaments should be nearly straight.

Different types of **connectors** link the filaments together (Fig. 1c). Each connector is composed of two filament-binding subunits, and acts as a mechanical link between two filaments of the network. Subunits differ in two ways: i) by the position on filaments to which they can bind; and

ii) by whether they can travel along the filament or not. We call a subunit that does not move a *binder,* and a subunit that moves a *motor*. "End-binders" are subunits that can only bind near the end of the filaments and do not move. 15 different types of connectors can be made from the 5 possible different subunits, and, for many of them, examples are found in living cells. For example, cross-linkers like alpha-actinin or filamin can be represented by a connector with identical binders that can bind anywhere along the filament; while bi-functional motors, such as members of the kinesin-5 family, Myosin IV motor proteins or dynein complexes are represented by connectors with two motor subunits that can bind anywhere along the filament. For simplicity, we assume that connectors do not interfere with each other, and that the subunits of connectors are non-interacting in the bound state. There is no limitation on the number of connectors that may bind to a filament. The motion of **motors** along a filament is unobstructed. Their velocity depends on force, but their detachment rate does not. A motor detaches immediately upon reaching the end of the filament on which it is travelling. A connector has no drag resistance and exerts opposite forces on the two points to which it is attached.

The system is characterized by a very low Reynolds number[1], and inertia of the object can be neglected. Moreover, we are here interested chiefly in the behavior of the system determined by the forces transmitted by the connectors and by the bending elasticity of the filaments. We thus focus on the regime where there are enough connectors to create a coherent mechanical ensemble. Filaments should be linked in such a way that the major factor that limits the extent to which forces are transmitted across the network is the filament bending elasticity, rather than the elasticity of the connections. We thus assumed that the connectors are short compared to the filaments, and remain so even when under tension. We find this the most interesting regime to study, since it should correspond to the one that is able to develop the strongest forces.

Because we intend to simulate thin networks such as the actin cortex underlying the plasma membrane, where the filaments are nearly parallel to the plasma membrane, all simulations were performed in 2D. We also ignored direct steric interactions and hydrodynamic coupling between the filaments. The motion of the filaments is thus determined by diffusion, and by constraints imposed by the connections between filaments. Under these assumptions, the microscopic motions of active connectors, which have a motor as one or both of their subunits, induce forces that lead to the overall deformation of the network. Passive connectors, although they do not generate forces themselves, have an essential role in transmitting the force generated by the motors. Our theory is based on the assumption that averaging over the many possible microscopic configurations of these elements will predict the bulk initial behavior of the entire system. We used Cytosim-based simulations[2] using the conditions described in this section, to test this prediction.

B. Description of the Simulations

The way in which a model network evolves was calculated using Cytosim[2], a software platform developed to model systems of flexible cytoskeletal fibers that are connected by different types of molecules. Cytosim is an Open Source project hosted on GITHUB (http://github.com/nedelec/cytosim). The algorithms of Cytosim efficiently solve the Brownian dynamics of the filaments, and the stochastic binding and unbinding of their associated molecules. Briefly, the Brownian dynamics of a point-like object is defined by an over-damped Langevin equation: $\xi \frac{dx}{dt} = f(x,t) + B(t)$, for a vector position x, where the right-hand terms are the deterministic and random forces respectively, and ξ is a drag coefficient typically calculated using Stokes' law from the viscosity of the fluid and the size of the object. Such an equation accurately

describes the motion of a micrometer-sized bead in a fluid that has the viscosity of cytoplasm. The filaments are elongated objects, discretized with "model-points" distributed regularly along their length (Fig. 3a). A large multivariate differential equation involving the coordinates of all the model-points is constructed and solved using a first-order implicit numerical integration scheme[2]. Although of higher dimensionality, this equation remains in essence similar to the equation presented here to model the motion of a single bead. In addition to Brownian motion in each point of the filament, the equation includes the bending elasticity of the filaments and the forces generated by the connectors (Fig. 3a). The implicit scheme makes it possible to integrate the system with a time-step of a few milliseconds, leading to a significant performance gain over explicit integration schemes, for which time steps possibly in the micro-second range must be used. Cytosim moreover uses algebraic constraints to ensure that the length of the filament remains constant, such that filaments are incompressible and inextensible, which seems physically appropriate for cytoskeletal filaments, considering the magnitude of the forces generated by molecular motors. For instance, the stretching elasticity of a single 1 µm-long actin filament was reported to be 35 pN/nm[3], corresponding to an elongation of 0.01% under the approximate force produced by one myosin motor.

Connectors are modeled in Cytosim as a point-like object with two independent filament-interacting elements, which correspond to the connector subunits in our theory. Each subunit can dynamically bind to and unbind from filaments, with predefined and constant rates (Fig. 3b,c,g). Binding and unbinding are first-order stochastic processes. The two subunits of a connector behave independently, except that they cannot both be bound to nearby positions on one filament. At any given time, some of the connectors are unbound, some are bound to only one filament, and some are bound to two filaments. Doubly bound connectors create Hookean springs with a zero resting-length. The associated stiffness (K) is a parameter of the simulation, and was set for this study sufficiently high to disallow the motors to extend significantly. Unbound connectors diffuse freely within the network, and may bind to any filament closer than their reach (ε) with a constant binding rate (k_{on}). Singly bound connectors unbind with a constant rate (k_{off}), and may bind to a second filament if it is within reach (ε), with the same rate (k_{on}). Doubly bound connectors may unbind from either subunit, doubling the effective unbinding rate ($2k_{off}$). In this work, the force that is present in the link of a doubly bound connector is not taken into account to calculate the unbinding rate. Under these simple assumptions, it is possible to predict the fraction of the connectors found in each configuration when equilibrium is reached (see part D). The force of a doubly bound motor reduces the speed of the motor linearly as $v = v_m\left(1 - \frac{f}{f_m}\right)$, where v_m is the unloaded speed of the motor, f_m is the stall force, and f is the component of the force parallel to the filament, taken to be positive if the force is antagonizing the spontaneous motion of the motor.

For all the simulations described in this study, the system was first equilibrated, both in terms of mechanical configuration of the filaments, and connector binding-unbinding kinetics for a sufficient time to reach equilibrium (typically 4 seconds of "simulated time"). During this time, the motors are not allowed to move (speed=0) and the system is passive. After equilibration, the motors are "activated", by setting the unloaded speed as desired, and the simulation is continued for a sufficient period of "simulated time" to estimate the network contraction rate.

C. Extraction of the Contraction Rate from Simulations

To be able to compare the performance of networks with different sets of parameters we determined a numerical value for the contraction rate. The contraction rate of the network is calculated as the difference in "network radius" divided by elapsed time. To estimate the network size, we first calculated the center of mass c as:

$$c = \frac{1}{P}\sum_i x_i$$

where the positions x_i of all the points used to represent filaments in the system are averaged (P is the total number of model points in the system). The size of the network is then calculated as:

$$R = \sqrt{\frac{2}{P}\sum_i (x_i - c)^2}$$

This is a simple and robust measure of network size, and if the filaments are uniformly distributed over a disc of radius Q, then $R \sim Q$. Thus, R is an indicator of the radius of the round area covered by a network, which contracts or expands isotropically (Appendix Fig S1).

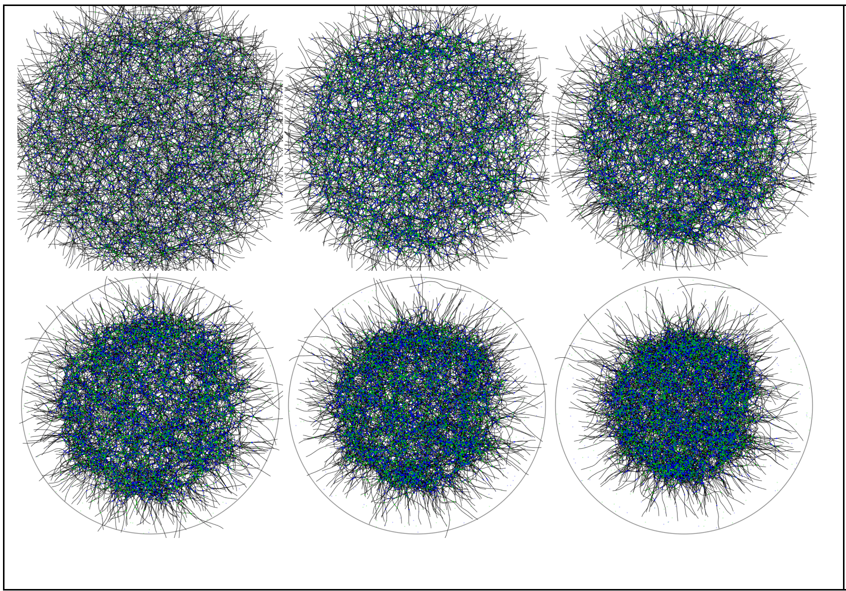

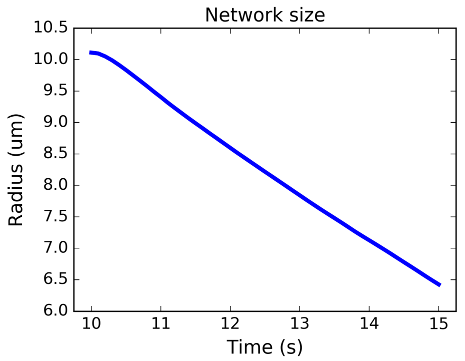

The radius as a function of time, estimated from the positions of the filaments. Here 10s were used for equilibration, during which the motors are not motile. At t=10s, the motors are activated and the network is simulated for another 5s.

Appendix Figure S1: Contraction of a network composed of 2000 filaments initially distributed over a disc of radius 10 μm. The length of the filaments is 5 μm, and the system contains 8141 motors and 9295 crosslinkers. Snapshots are separated by intervals of 1s. The extracted size of the network, as defined by the formula described above, is plotted on the right. The radius as a function of time is nearly linear, and the contraction rate (the slope) is well defined.

The contraction rate of the network, which has units of μm/s, is then simply estimated as a finite difference between two time points:

$$\frac{dR}{dt} = \frac{R(t_2) - R(t_1)}{t_2 - t_1}$$

For all simulations, we allow some relaxation time before calculating the contraction rate. The contraction rate diminishes exponentially (data not shown), and we calculated it over a period of 5 seconds over which it is stable. Negative values of dR/dt indicate that the system is contracting, while positive values reflect expansion.

D. Calculation of the Connector Subunits Probabilities

The calculation of the network response is based on the probability of finding at least one or more connector subunits at each filament crossing. This involves an estimation of the number of filament crossings and the number of doubly bound connectors in the network.

To calculate the number of filament crossings we consider here that the network occupies a two-dimensional disc of surface S, and is made of F filaments that all have the same length L. For

simplicity, we assume that $L \ll \sqrt{S}$, and that filaments are segments of lines positioned randomly within the disc. For a pair of segments forming an angle θ, the probability of them intersecting is $\frac{L^2}{S}\sin\theta$, and the total number of intersections is therefore:

$$X = \frac{F(F-1)L^2}{\pi S}$$

Henceforth, the average number of intersections per filament is $2X/F$.

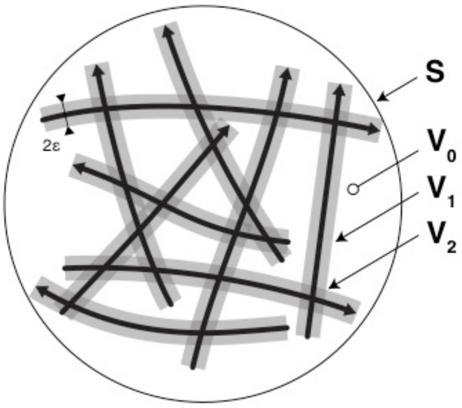

Appendix Figure S2: Regions within the network that can capture connectors. The surface area S can be partitioned into three regions: a region V_0 from which a connector cannot bind, because all filaments are further than the maximum binding distance ε, a region V_1 from which a connector can bind to one filament, but not to two, and a region V_2 from which a connector may link two filaments.

In Cytosim, unbound connectors diffuse freely within the disc that contains the filaments, and we assume that their distribution remains uniform, which should be the case if the associated diffusion constant is high enough. Considering the distance ε at which subunits are able to bind, we partition the surface as follows (Appendix Fig S2): a region V_0 from which binding cannot occur, because there is no filament closer than ε. A region V_1 where binding can lead to a single attachment only, because only one filament is within binding distance ε, and a region V_2 located near an intersection where binding can lead to a connection between two filaments. The size of the three regions can be calculated as $V_2 = 4\pi X\varepsilon^2$ and $V_1 + V_2 = 2FL\varepsilon$, assuming that ε is small such that $2FL\varepsilon \ll S$. To calculate V_2, we integrated over all possible intersection angles. Given these quantities, the binding and unbinding rates (k_{on}, k_{off}), define the transition rates in the state diagram (Appendix Fig S3).

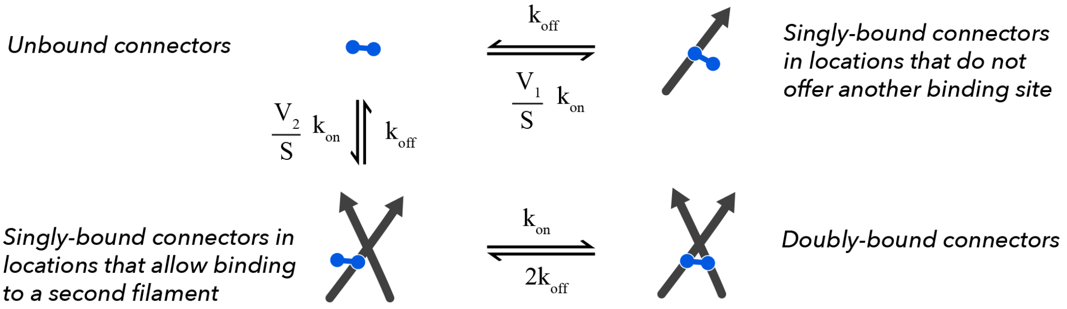

Appendix Figure S3: Reaction diagram for connectors within the network. The effective transition rates are defined by the different partitions of the system volume, and the molecular binding and unbinding rates of the activities that make up a connector.

Finding the equilibrium quantities for such a system (Appendix Fig S3) yields in particular the number of doubly bound connectors. For example, if the system contains M motor entities, the number of doubly bound motors of this type will be:

$$M_2 = M\frac{a^2}{2}\frac{V_2}{S}\left(1 + a\frac{V_1 + V_2}{S} + \frac{a^2}{2}\frac{V_2}{S}\right)^{-1}$$

with

$$a = \frac{k_{on}}{k_{off}}$$

The quantity M_2 corresponds to the average number of motors in the entire network. To calculate the statistical distribution of motor on an intersection, we will not consider the angle at which filaments intersect and with this simplification we expect the connectors to be distributed uniformly over all intersections. In this case, the number of connectors of each type on a single intersection is expected to follow a Poisson law, characterized by a single parameter λ (the mean occupancy). The probability of having k connectors at a single intersection is:

$$P(k) = \lambda^k \frac{e^{-\lambda}}{k!}$$

Hence, $P(0) = e^{-\lambda}$ is the probability of having no connector on a particular intersection, $P(1)$ the probability of having exactly one connector, and so on. Different subunits will have different parameters depending on the number of doubly bound connectors associated with each subunit. For example, in the case of the actomyosin system there are two mean occupancy parameters:

$$\lambda_M = \frac{M_2}{X}; \qquad \lambda_C = \frac{C_2}{X}$$

This will define the probabilities of finding at least one or more motors or one or more crosslinkers per filament intersections:

$$P_M = 1 - e^{-\lambda_M}; \qquad P_C = 1 - e^{-\lambda_C}$$

In our calculations, we will derive contractility from P_M and P_C only, ignoring the higher order terms of the distributions. This means that we will only take into account whether an intersection has a certain type of connector or not, but we will not distinguish whether it has one or two (or more) of this type.

E. Prediction of Contraction Rates for Various Connector Mixtures

The qualitative predictions on the horizontal axis of Fig. 5c are calculated as the weighted sums of all expansile and contractile configurations ($\sum_i p_i v_i$), where p_i is the probability of each configuration to occur and $v_i = \frac{da}{dt}$ is the relative movement of the subunits involved in each configuration. For this figure, we only considered the two limit cases: the regime where filaments are straight and the regime where they buckle under any compressive force. Since all pushing (expansile) configurations have positive values of v_i, while pulling (contractile) configurations have negative values, the sign of the net sum indicates the predicted network outcome (negative, contractile; positive, expansile).

The probability for each configuration was calculated in the same manner as described for the example in the main text for the actin system. For each pair of subunits along a filament we calculate the probability of finding each subunit at a given filament crossing times the probability of not finding a subunit that may cancel the action of the first. For example, a plus-end motor can only be effective if there is not a minus-end motor or a binder subunit (either a general or a minus end-binder) at the same intersection. Thus, a configuration with two opposite motors moving toward each other has the following probability:

$$P_+(1-P_-)(1-P_o)(1-P_m) \times P_-(1-P_+)(1-P_o)\big(1-P_p\big)$$

where P_+, P_-, P_o, P_p and P_m are the probabilities of having at least one plus-end motor, minus-end motor, generic binder, plus-end binder or minus-end binder subunit per filament crossing, respectively; and $1-P$ is the probability of not having that particular subunit. These probabilities are associated with the subunits, and are obtained by summing the connector probabilities calculated as in section D2, taking into account the composition of each connector. For example, a connector composed of two motor (e.g. ++) contributes with a coefficient 1 to the mean occupancy parameter λ_+ used to calculate the probability of its subunits (P_+). A heterogeneous connector composed of different subunits (e.g. +m) contributes with a coefficient $^1/_2$ to their respective occupancy parameters (λ_+ and λ_m).

F. Prediction of the Contraction/Expansion Rate with End-Binders

We derive here the prediction for the network presented on Figure 4, which is plotted with a dashed line on Figure 4d. This 2D network is composed of one kind of plus-end directed motor (+), and two types of end-binders (m, p) from which two types of connectors are made: (+p) and (+m). We first calculate the number of connectors and the probabilities of the active configurations involving two connectors. From this, we derive the net effects of all configurations, which when negative (resp. positive) predicts a contractile (resp. expansile) behavior.

Given δ, the size of the region near the filament-end (minus- or plus- end) to which an end-binder can attach (Fig. 3d), and following the arguments of section D, the number of intersections occurring near the end of a filament, and at any position of another filament is:

$$X_\mathrm{p} = X_\mathrm{m} = \frac{F(F-1)}{\pi S} L\delta$$

To calculate the equilibrium number of connectors that bridge two filaments we partition the volume as on Figure S2, replacing X by $X' \in [X_\mathrm{m}, X_\mathrm{p}]$ and defining $V_2 = 4\pi X'\varepsilon^2$ and $V_1 + V_2 = 2FL\varepsilon$. The binding rate is also modified since the end-binder may not bind to most of V_1, assuming that $\delta \ll F$. The quantities of connectors in the different states are calculated assuming that equilibrium is reached as on Figure S3. Following this approach, we calculate the expected mean number of doubly bound connectors: m_2 and p_2 (both quantities are positive). In the simulation, the number of connectors of each type on a single intersection is expected to follow a Poisson distribution:

$$P(k) = \lambda^k \, \frac{e^{-\lambda}}{k!}$$

Where $P(k)$ is the probability of having k connectors at a single intersection and λ is a parameter related to the number of doubly bound connectors of each type:

$$\lambda_\mathrm{m} = \frac{1}{2}\frac{m_2}{X_\mathrm{m}}; \qquad \lambda_\mathrm{p} = \frac{1}{2}\frac{p_2}{X_\mathrm{p}},$$

from which we derive:

$$P_\mathrm{m} = 1 - e^{-\lambda_\mathrm{m}}; \qquad P_\mathrm{p} = 1 - e^{-\lambda_\mathrm{p}}$$

The system has two active configurations, involving one motor and one end binder (Fig. 4a). The likelihood of these configurations is P_+P_m and P_+P_p, respectively. Because both connectors in the system involve identical motor subunits, one calculates $P_+ = 1 - e^{-\lambda_+}$ from $\lambda_+ = \frac{1}{2}(m_2 + p_2)/(X_\mathrm{m} + X_\mathrm{p})$. Finally, the sum of the contributions of all active configurations weighted by the relative subunit movements yields:

$$S = \sum_i v_i p_i = vP_+P_\mathrm{m} - vP_+P_\mathrm{p} = v(1 - e^{-\lambda_+})(e^{-\lambda_\mathrm{p}} - e^{-\lambda_\mathrm{m}})$$

The sign of S is determined by the factor $(e^{-\lambda_\mathrm{p}} - e^{-\lambda_\mathrm{m}})$ and predicts whether the system is net contractile or net extensile. The system is extensile if $p_2 = 0$ and contractile if $m_2 = 0$. For the cases where both the minus-end-binder and the plus-end binder subunits have the same binding/unbinding parameters, the system is symmetric, and extensile when the number of (+m) is higher than the number of (+p), neutral if $p_2 = m_2$, and contractile otherwise (Fig. 4d,e).

G. Contraction Rate for Networks of Semi-Flexible Filaments

For the actomyosin system show in Fig. 2, the theory predicts that the network response should be neutral if the filaments are rigid and net contractile if they are flexible or semi-flexible, as is the case for actin filament. The amount of contraction, therefore, should scale with the propensity of filaments to buckle.

Generally, the distance between the two connectors of a configurations can be expressed as βL_1, where L_1 is the network mesh-size and β is a continuous parameter. In simple terms, $\beta - 1$ is the number of filament intersections located between the two connectors. For any given β, the probabilities of the contractile and expansile configurations are equal, and thus in the absence of buckling, pulling and pushing cancel each other out, producing no net change in network size. However, there exists a threshold β_0 above which buckling spoils the expansile forces, and thus the configurations with $\beta > \beta_0$ can lead to net contraction. This is illustrated in Figure S4, for integer values of β.

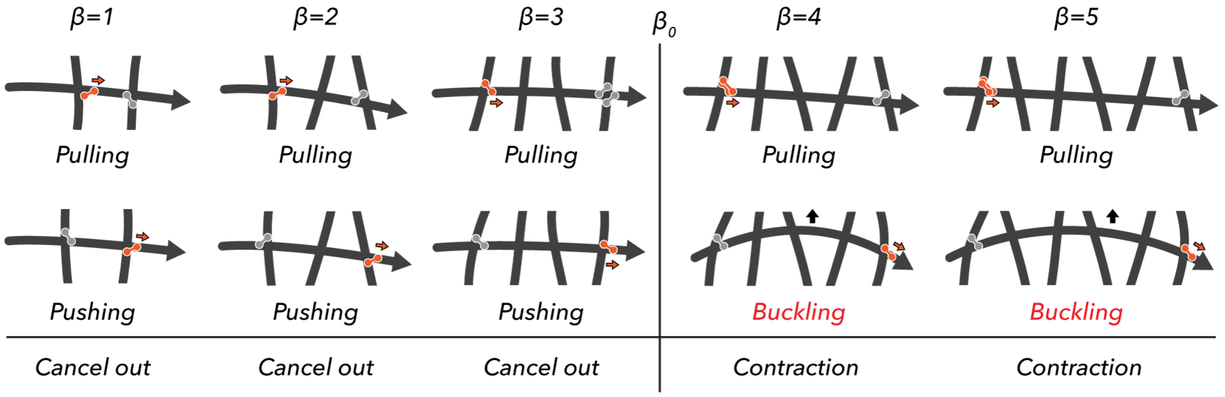

Appendix Figure S4: Configurations with two connectors found in a random network. The top row contains pulling configurations, while the bottom row depicts the pushing configurations obtained by swapping motor and crosslinker. The probability of a (top) pulling configuration is thus always equal to the corresponding (bottom) pushing configuration. From left to right, the connectors are positioned increasingly far apart, separated by a distance βL_1, which is a multiple of the network mesh-size L_1. Note that intermediate filament crossings are unconnected, and do not affect the mechanics of the configurations here. However, β determines the likelihood of the configuration, and the buckling force of the filament. For a certain value β_0, the distance $\beta_0 L_1$ permits filament buckling. The value of β_0 depends on filament rigidity and the forces of the motors, and on this illustration, $\beta_0 = 3.5$. For $\beta < \beta_0$, pushing and pulling contributions cancel out. For $\beta > \beta_0$, pulling and pushing do not cancel out since buckling spoils the pushing contributions, leading instead to net contraction.

The likelihood of a configuration in which two connectors are separated by βL_1 is $P_M(1 - P_C)^\beta P_C$, where L_1 is the mesh size and $\beta - 1$ is the number of intermediate unconnected filaments (Appendix Fig S5). Since, with all other things being kept equal, this likelihood decreases with β, shorter configurations are always more abundant than longer ones. Thus, generally, one

would expect the smallest β that allows buckling to correspond to the configuration that has the highest impact on the network.

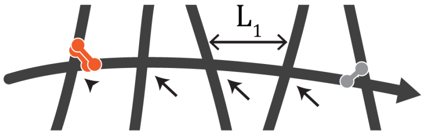

Appendix Figure S5: The probability of a configuration depends on its length parameter β, and can be obtained by multiplying the likelihoods associated with each intersection. The first intersection carries a probability $P_M(1-P_C)$ since it should have at least one motor (P_M) and no crosslinker $(1-P_C)$. The last intersection on the right carries a probability P_C, since it should have at least one crosslinker, but it may or may not have a motor. By definition of the parameter β, the intermediate intersections indicated with arrows are free of crosslinkers, and if their number is $\beta-1$ (here, $\beta=4$), this occurs with a probability $(1-P_C)^{\beta-1}$. Finally, the entire configuration has a probability $P_M(1-P_C)^{\beta}P_C$. Note that it is not specified if the intermediate positions have a motor or not.

Finally, the threshold β_0 can be estimated from the density of the network, the stall force of the motors and the bending rigidity of the filaments, by equalizing the force exerted by the motor f_{motor} with the buckling threshold of the filament over the length $\beta_0 L_1$. We use here $f_{Euler}=\frac{4\pi^2\kappa}{(\beta_0 L_1)^2}$, given the bending rigidity of the filament κ, and assuming that the filament cannot freely rotate at the ends (because it has more connections). We thus derive:

$$\beta_0=\frac{2\pi}{L_1}\sqrt{\frac{\kappa}{f_{motor}}}$$

This estimate assumes that only one motor is acting at the intersection, which is not always the case. Despite these potential complications, we find that this value of β_0 provides accurate fits. The likelihood of the shortest pulling configuration (Appendix Figs S5) varies like the numerator of χ (Box 1B) while P_M and P_C change (and are fully determined by the parameters of the system). It corresponds to $\sum_i \phi\, p_i v_i$, the numerator of the fraction defining χ (Box 1B) because in this sum there is only one term with $\phi\, v_i<0$. It is sufficient here to consider the numerator, since the denominator does not vary as the number of motors is changed. Hence taking away the constant terms lead to the fitting function:

$$f=\ P_M(1-P_C)^{\beta_0}P_C$$

Given the value β_0, a scaling factor γ between f and the contractile rate can be calculated directly, since the best fit to a series of simulation data points $\{g_i\}$ is $\{\gamma f_i\}$ with $\gamma=\sum f_i\, g_i/\sum {f_i}^2$, to minimize the sum of the squared residues. In practice, the values $\{f_i\}$ are calculated for each data point from the simulation parameters used to produce $\{g_i\}$.

Appendix Figure S6: Contraction of a Network of Semi-Flexible Filaments (data as shown on Figure 2C)

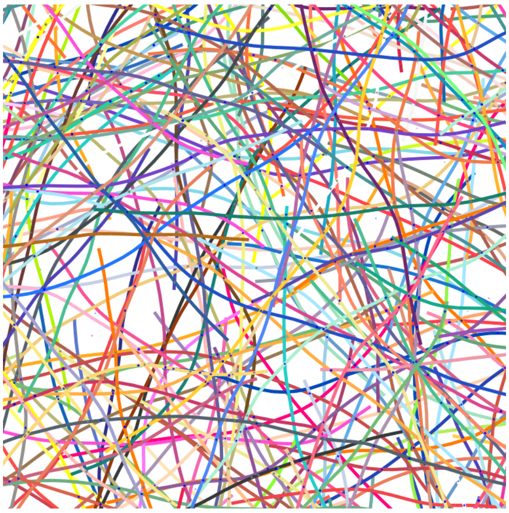

A 2×2μm portion of a network composed of 2000 filaments of length 5μm, contained within a circle of radius 10μm. The flexibility of the filament is similar to F-actin (0.05 pN×μm^2). The mesh size is 55nm and at this density, there are ~90 intersections per filament.

The predicted exponent is $\beta = 10.43$.

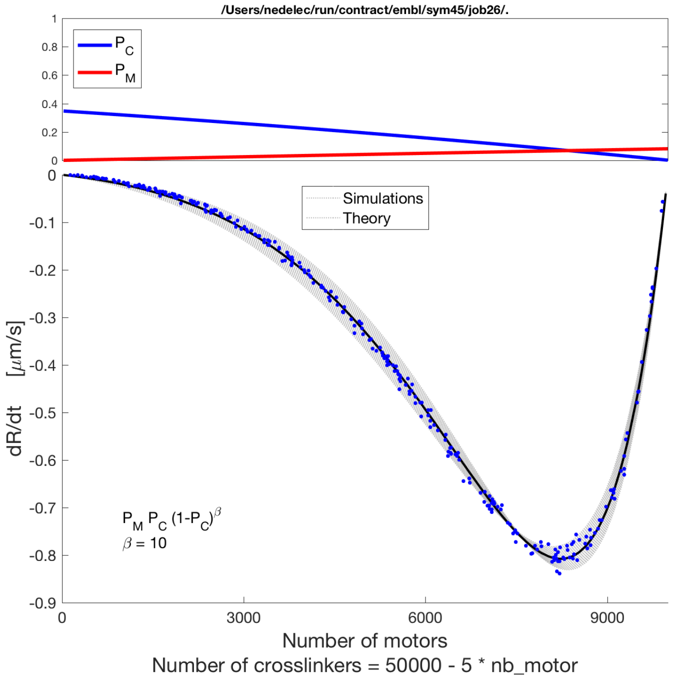

Networks are simulated with varying numbers of crosslinkers, while the number of motors and crosslinkers are varied inversely. The resulting contraction rate is fitted by a single probability function associated with the dominant contractile configuration. The fit is obtained here with $\beta = 10$. The grey shaded area indicates the fits obtained with the range of values $\beta = [9, 11]$.

Appendix Table S1 – Simulation Parameters

This table lists the parameters of the simulation. Whenever possible, we used published, experimentally determined values.

Name	Value	Note
Time step	1 millisecond	Computational parameter. Total time simulated ~ 10s
Viscosity	0.1 pN s/µm²	Effective viscosity of the fluid
k_BT	0.0042 pN µm	Thermal energy at 25°C, defining the Brownian motion of the filaments
Network geometry	R = 15 to 25 µm	Radius of the circular geometry
**Filaments**		
Filament length	5 µm	[4]
Filament rigidity	0.01 pN µm²	For flexible filaments (Fig. 2) [5]
	0.075 pN µm²	For actin-like system (Fig. 6) [1,6]
	infinite	Rigid filaments are modelled with only one segment, and may not bend.
Filament segmentation	Between 0.1 and 0.2 µm	Computational parameter
**Motor subunits**		
Binding	range 10 nm<br>rate 10 s^{-1}	Maximal distance from which a motor can bind to a filament, and rate at which binding can occur
Unbinding	rate 0.3 s^{-1}	Unbinding is independent of load
Motility	Unloaded speed: $v_m = 0.2\ \mu m/s$<br>Stall force: $f_s = 6\ pN$	The velocity of a motor varies with force $\vec{f}$, as: $v = v_m(1 + \vec{f} \cdot \vec{d}/f_s)$, where $\vec{d}$ is the direction in which the motor would move along the filament if it was unloaded.
**Binder subunits**		
Binding	range 10 nm<br>rate 10 s^{-1}	Maximal distance from which a binder can bind to a filament, and rate at which binding occurs
Unbinding	rate 0.3 s^{-1}	Unbinding is independent of load
End-binding length	δ = 0.5 µm	Size of region near the plus- or minus-end to which a 'end-binder' may attach
**Couples**		
Link stiffness	k = 500 pN/µm	Stiffness of the Hookean link between the two subunits of a Couple. If the separation is $\vec{u}$, the force is $\vec{f} = k\vec{u}$
Diffusion	D = 100 µm²/s	

Appendix Table S2 - Biological Parameters

This table lists parameter values measured *in vivo* and *in vitro*.

Name	Value	Reference
**Medium viscosity**		
Water	0.001 pN s/μm²	
D. melanogaster cytosol	~0.3 pN s/μm²	[2,7]
C. elegans cytosol	~1 pN s/μm²	
Cleared cytoplasm	0.02 pN s/μm²	
**F-Actin**		
Length *in vitro*	0–10 μm	[2,4]
Rigidity	0.075 pN μm²	[2,5]
**Microtubules**		
Rigidity	22 pN μm²	[3,5]
**Crosslinkers**		
α-actinin unbinding rate	0.37-3.2 s^{-1}	[4,8]
	0.4 s^{-1}	[5,6,9]
	5-15 s^{-1}	[10]
	0.3-0.4 s^{-1}	[11]
Filamin unbinding rate	0.6 s^{-1}	[9]
Fascin unbinding rate	0.12 s^{-1}	[12]
**Myosin**		
Binding rate	0.5-1 s^{-1}	[13]
	6 s^{-1}	[14]
Unbinding rate	0.18 s^{-1}	[15]
	13-15 s^{-1}	[14]
Speed	0.04–1.7 μm/s	[16]
	0.3 μm/s	[15]
	0.01–5 μm/s	[17]
Stall force	2.2 pN	[18]
	4-12 pN	[19]
**Dynein**		
Unbinding rate	0.667 s^{-1}	[20,21]
Speed	0.8 μm/s	[22]
Stall force	7-8 pN	[22]
**Kinesin**		
Speed	0.6-0.7 μm/s	[23]
Unbinding rate	0.314 s^{-1}	[20,21]
Stall force	5-6 pN	[23]

Appendix Table S3 - Cytosim Configuration File

```
% This file was used to make Figure 2C
% It is a templated configuration file
% that must be processed by Preconfig:
% www.github.com/nedelec/preconfig

set simul contract
{
    time_step = 0.001
    viscosity = 0.1
}

set space cell
{
    geometry = circle 10
}
new space cell

set fiber filament
{
    rigidity = 0.05
    segmentation = 0.1
}

set hand binder
{
    binding_rate = 10
    binding_range = 0.01
    unbinding_rate = 0.5
    unbinding_force = inf
}

set hand plus_motor
{
    binding_rate = 10
    binding_range = 0.01
    unbinding_rate = 0.5
    unbinding_force = inf
    activity = move
    max_speed = 0
    stall_force = 6
}

set couple crosslinks
{
    hand1 = binder
    hand2 = binder
    stiffness = 250
    diffusion = 100
}

set couple motor
{
    hand1 = plus_motor
    hand2 = plus_motor
    stiffness = 250
    diffusion = 100
}

new 2000 fiber filament
{
    length = 5
}

% let the shape of filaments equilibrate
% from their Brownian motion:
run 5000 simul *
{
    solve = 1
}

% the bracketed templated values
% will be replaced by Preconfig:
[[motor = random.randint(0, 10000)]]

new [[motor]] couple motor
new [[50000-5*motor]] couple crosslinks

% equilibrate the binding/unbinding
% of connectors while filaments are
% not allowed to move:
run 25000 simul *
{
    solve = 0
}

% activate the motors:
change hand plus_motor
{
    max_speed = 0.5
}

% simulate 5000 steps and save 5 frames:
run 5000 simul *
{
    nb_frames = 5
}
```

References

1. Purcell, E. M. Life at low Reynolds number. *Am J Phys* **45,** 3–11 (1977).
2. Nedelec, F. & Foethke, D. Collective Langevin dynamics of flexible cytoskeletal fibers. *New Journal of Physics* **9,** 499–510 (2007).
3. Liu, X. & Pollack, G. H. Mechanics of F-Actin Characterized with Microfabricated Cantilevers. *Biophysical journal* **83,** 2705–2715 (2002).
4. Burlacu, S., Janmey, P. A. & Borejdo, J. Distribution of actin filament lengths measured by fluorescence microscopy. *Am. J. Physiol.* **262,** C569–77 (1992).
5. Gittes, F., Mickey, B., Nettleton, J. & Howard, J. Flexural rigidity of microtubules and actin filaments measured from thermal fluctuations in shape. *J Cell Biol* **120,** 923–934 (1993).
6. Gittes, F., Mickey, B., Nettleton, J. & Howard, J. Flexural rigidity of microtubules and actin filaments measured from thermal fluctuations in shape. *J Cell Biol* **120,** 923–934 (1993).
7. Polyakov, O. Y. *Mechanical aspects of Drosophila gastrulation*. (2013).
8. Wachsstock, D. H., Schwartz, W. H. & Pollard, T. D. Affinity of alpha-actinin for actin determines the structure and mechanical properties of actin filament gels. *Biophys J* **65,** 205–214 (1993).
9. Goldmann, W. H. & Isenberg, G. Analysis of filamin and alpha-actinin binding to actin by the stopped flow method. *FEBS Lett* **336,** 408–410 (1993).
10. Miller, B. M., Nyitrai, M., Bernstein, S. I. & Geeves, M. A. Kinetic analysis of Drosophila muscle myosin isoforms suggests a novel mode of mechanochemical coupling. *J Biol Chem* **278,** 50293–50300 (2003).
11. Fritzsche, M., Lewalle, A., Duke, T., Kruse, K. & Charras, G. Analysis of turnover dynamics of the submembranous actin cortex. *Molecular biology of …* (2013).
12. Aratyn, Y. S., Schaus, T. E., Taylor, E. W. & Borisy, G. G. Intrinsic dynamic behavior of fascin in filopodia. *Mol Biol Cell* **18,** 3928–3940 (2007).
13. Rovner, A. S., Fagnant, P. M. & Trybus, K. M. Phosphorylation of a Single Head of Smooth Muscle Myosin Activates the Whole Molecule †. *Biochemistry* **45,** 5280–5289 (2006).
14. Guo, B. & Guilford, W. H. Mechanics of actomyosin bonds in different nucleotide states are tuned to muscle contraction. *Proc Natl Acad Sci USA* **103,** 9844–9849 (2006).
15. Mehta, A. D. *et al.* Myosin-V is a processive actin-based motor. *Nature* **400,** 590–593 (1999).
16. Finer, J. T., Simmons, R. M. & Spudich, J. A. Single myosin molecule mechanics: piconewton forces and nanometre steps. *Nature* (1994).
17. Barua, B., Nagy, A., Sellers, J. R. & Hitchcock-DeGregori, S. E. Regulation of nonmuscle myosin II by tropomyosin. *Biochemistry* **53,** 4015–4024 (2014).
18. Norstrom, M. F., Smithback, P. A. & Rock, R. S. Unconventional processive mechanics of non-muscle myosin IIB. *J Biol Chem* **285,** 26326–26334 (2010).
19. Walcott, S., Warshaw, D. M. & Debold, E. P. Mechanical coupling between myosin molecules causes differences between ensemble and single-molecule measurements. *Biophys J* **103,** 501–510 (2012).
20. Soppina, V., Rai, A. & Mallik, R. Simple non-fluorescent polarity labeling of microtubules for molecular motor assays. *Biotechniques* **46,** 543–549 (2009).
21. Bhat, D. & Gopalakrishnan, M. Effectiveness of a dynein team in a tug of war helped by reduced load sensitivity of detachment: evidence from the study of bidirectional endosome transport in D. discoideum. *Phys Biol* **9,** 046003 (2012).
22. Toba, S., Watanabe, T. M., Yamaguchi-Okimoto, L., Toyoshima, Y. Y. & Higuchi, H. Overlapping hand-over-hand mechanism of single molecular motility of cytoplasmic dynein. *Proc Natl Acad Sci U S A* **103,** 5741–5745 (2006).
23. Svoboda, K. & Block, S. M. Force and velocity measured for single kinesin molecules. *Cell* **77,** 773–784 (1994).
