# Supplementary material for: A theory that predicts behaviors of disordered cytoskeletal networks
Source: Mol Syst Biol. 2017 Sep 27;13(9):941. doi: 10.15252/msb.20177796 (PMC5615920; doi:10.15252/msb.20177796)
Supplement: Supplementary file 2 — Movie EV1 [file MSB-13-941-s002.zip › MSB_7796_movielegend_EV1.docx]

MOVIE LEGEND

**Movie EV1**

Contraction of a network of 1000 filaments of length 5 µm (red) distributed over a disc of radius 10 µm, with 8000 motors (blue) and 8393 crosslinkers (gray). This movie is similar to the network shown on Figure 2B, but the filament rigidity is 0.05 pNµm^2^, and the segmentation is 50nm.
