# Supplementary material for: A theory that predicts behaviors of disordered cytoskeletal networks
Source: Mol Syst Biol. 2017 Sep 27;13(9):941. doi: 10.15252/msb.20177796 (PMC5615920; doi:10.15252/msb.20177796)
Supplement: Supplementary file 3 — Movie EV2 [file MSB-13-941-s003.zip › MSB_7796_movielegend_EV2.docx]

MOVIE LEGEND

**Movie EV2**

Detailed view of part of the network shown on movie 1. A few (5%) randomly chosen filaments are drawn here in pink, while the others are depicted in dark red. Contraction of the network is accompanied by the buckling of all filaments.
