# Supplementary material for: A theory that predicts behaviors of disordered cytoskeletal networks
Source: Mol Syst Biol. 2017 Sep 27;13(9):941. doi: 10.15252/msb.20177796 (PMC5615920; doi:10.15252/msb.20177796)
Supplement: Supplementary file 4 — Movie EV3 [file MSB-13-941-s004.zip › MSB_7796_movielegend_EV3.docx]

MOVIE LEGEND

**Movie EV3**

Contraction of a network of 1000 filaments of length 2.5 µm distributed over a disc of radius 5 µm, with 8000 motors and 8393 crosslinkers. Filament rigidity is 0.05 pNµm^2^, and segmentation is 50nm. The motors and crosslinkers are not shown. Filaments are drawn with color shades indicating their local axial tensions, ranging from dark blue shades indicating low tension to red indicating the highest tension. Yellow corresponds to tension of about 10 pN.
