# Supplementary material for: A theory that predicts behaviors of disordered cytoskeletal networks
Source: Mol Syst Biol. 2017 Sep 27;13(9):941. doi: 10.15252/msb.20177796 (PMC5615920; doi:10.15252/msb.20177796)
Supplement: Supplementary file 5 — Movie EV4 [file MSB-13-941-s005.zip › MSB_7796_movielegend_EV4.docx]

MOVIE LEGEND

**Movie EV4**

Evolution over time of 16 examples of networks of flexible filaments shown in Figure 2D. The number of crosslinkers is varied from left to right (750, 3000, 12000 and 48000), and the number of motors is varied from bottom to up (750, 3000, 12000 and 48000). All simulations start with 1500 straight filaments distributed over a circular area of radius 15 μm. The timespan covered is 30s.
