# Supplementary material for: A theory that predicts behaviors of disordered cytoskeletal networks
Source: Mol Syst Biol. 2017 Sep 27;13(9):941. doi: 10.15252/msb.20177796 (PMC5615920; doi:10.15252/msb.20177796)
Supplement: Supplementary file 6 — Movie EV5 [file MSB-13-941-s006.zip › MSB_7796_movielegend_EV5.docx]

MOVIE LEGEND

**Movie EV5**

Evolution over time of the network shown in Figure 3B. Network composed of 1500 straight (*i.e.* with infinite rigidity) filaments (white), 750 motor-plus-end-binders (red) and 48000 motor-minus-end-binders (blue) connectors, distributed over a circular area of radius 15μm. The higher proportion of motor-minus-end-binders connectors leads to a higher probability of extensile configurations where filaments are pushed apart. The timespan covered is 30s.
