# Supplementary material for: A theory that predicts behaviors of disordered cytoskeletal networks
Source: Mol Syst Biol. 2017 Sep 27;13(9):941. doi: 10.15252/msb.20177796 (PMC5615920; doi:10.15252/msb.20177796)
Supplement: Supplementary file 7 — Movie EV6 [file MSB-13-941-s007.zip › MSB_7796_movielegend_EV6.docx]

MOVIE LEGEND

**Movie EV6**

Movie of the network shown in Figure 3C which is composed of 1500 straight (*i.e.* with infinite rigidity) filaments (white), 48000 motor-plus-end-binders (red) and 750 motor-minus-end-binders (blue) connectors distributed over a circular area of radius 15μm. The higher proportion of motor-plus-end-binders connectors leads to a higher probability of contractile configurations where filaments are pulled together towards their plus ends. The timespan covered is 60s.
