# Supplementary material for: A theory that predicts behaviors of disordered cytoskeletal networks
Source: Mol Syst Biol. 2017 Sep 27;13(9):941. doi: 10.15252/msb.20177796 (PMC5615920; doi:10.15252/msb.20177796)
Supplement: Supplementary file 8 — Movie EV7 [file MSB-13-941-s008.zip › MSB_7796_movielegend_EV7.docx]

MOVIE LEGEND

**Movie EV7**

Evolution over time of different networks of straight filaments (*i.e.* with infinite rigidity) as shown in Figure 3E. The number of motor-minus-end-binders is varied from bottom to top (750, 3000, 12000 and 48000), and the number of motor-plus-end-binders per filament is varied from left to right (750, 3000, 12000 and 48000). All simulations start with 1500 straight filaments and a varying number of connectors randomly distributed over a circular area of radius 15 µm. The timespan covered is 30s.
