# Supplementary material for: A theory that predicts behaviors of disordered cytoskeletal networks
Source: Mol Syst Biol. 2017 Sep 27;13(9):941. doi: 10.15252/msb.20177796 (PMC5615920; doi:10.15252/msb.20177796)
Supplement: Supplementary file 9 — Movie EV8 [file MSB-13-941-s009.zip › MSB_7796_movielegend_EV8.docx]

MOVIE LEGEND

**Movie EV8**

Network with filament turnover (lifetime ~11.11s) and periodic boundary conditions (size 29x29 µm). The network contains 10000 flexible filaments of length 2.5µm (rigidity 0.075 pN µm^2^), 80000 motors, 40000 crosslinkers.

Foci move and fuse with each other.
